# Clinical and radiographic evaluation of indirect pulp treatment of young permanent molars using photo-activated oral disinfection versus calcium hydroxide: a randomized controlled pilot trial

**DOI:** 10.1038/s41405-020-0030-z

**Published:** 2020-03-17

**Authors:** Marwa Aly Elchaghaby, Dalia Mohamed Moheb, Osama Ibrahim El Shahawy, Ahmed Mohamed Abd Alsamad, Mervat Abdel Moniem Rashed

**Affiliations:** Faculty of Dentistry, Cairo University, Cairo, Egypt

**Keywords:** Paediatric dentistry, Oral diseases

## Abstract

**Background:**

Calcium hydroxide is the most commonly used material in indirect pulp treatment (IPT). However, its drawbacks required its replacement by other materials.

**Aim:**

This study aims to estimate clinically and radiographically the success of indirect pulp treatment of young permanent molars with either photo-activated oral disinfection (PAD) or calcium hydroxide.

**Design:**

This Randomized Controlled Pilot Trial included 32 vital first permanent molars with deep caries that were treated by indirect pulp treatment with either PAD (group 1) or calcium hydroxide (group 2). Clinical and radiographic success in addition to newly-formed dentin thickness were evaluated regularly at 2, 6, 9, and 12 months.

**Results:**

The success for both groups was 100% clinically and radiographically at all follow-up periods. Regarding the mean thickness of newly-formed dentin for both groups at different follow-up periods, there was no statistically significant difference between both groups at 2, 6, 9, and 12 months, with *P* values = 0.825, 0.146, 0.280, and 0.400, respectively.

**Conclusions:**

The clinical and radiographic success for indirect pulp treatment of young permanent molars with both PAD and calcium hydroxide were comparable.

## Background

Indirect pulp treatment is a procedure, in which pulp exposure is prevented by preserving the carious dentin bordering the pulp and sealing the pulp with a biocompatible material.^1^ Calcium hydroxide is the gold standard for pulp capping. It maintains the pulp vital, allows reparative dentin formation, shelters the pulp against harmful stimuli and has antimicrobial effect.^2^ However, many disadvantages were reported with its use over time including poor seal, lack of chemical and mechanical adhesion, poor strength, long-term solubility, enhanced disintegration after acid etching, and tunnel defects in the dentin bridge.^3^ The operative practice is to excavate the soft dentin to eradicate infected tissue; however, it is difficult to eliminate all the microorganisms because few will remain even though the soft dentin was removed.^4^

Evidence suggests that for arresting caries lesions, it is not crucial to remove the infected dentin entirely and that selective caries removal and composite restoration can yield better clinical results.^5^ For more conservative and effective treatment, disinfection instead of complete caries removal has been encouraged.^6^ Photo-activated oral disinfection (PAD) is a technology employing two nontoxic components, a photo-activating liquid, and a LED light source. The LED light source specifically targets the cariogenic bacteria and periodontal pathogens.^7^ The usage of PAD in caries management can eradicate remaining bacteria in soft dentin, reassures rapid healing and improve the prognosis of treatment.^8^

Therefore, this study aims to estimate clinically and radiographically the success of PAD and calcium hydroxide in indirect pulp treatment (IPT) of young permanent molars. This study adopted the Null Hypothesis, that there is no difference between the success of PAD and calcium hydroxide in IPT of young permanent molars.

## Materials and methods

### Trial design

This study is a randomized controlled pilot trial with two parallel groups that included thirty-two vital deep carious lower first permanent molars in healthy cooperative children with age range (6–12 years old). The Research Ethics Committee, Faculty of Dentistry—Cairo University approved the study protocol. This trial has been registered with clinicaltrials.gov under the title: Indirect pulp treatment of young permanent molars using photo-activated oral disinfection vs. calcium hydroxide with registration number NCT03631277.

### Sample size calculation

Sample size was determined to yield statistically significant results for the measurement of the thickness of newly formed dentin in each group. It was estimated based on ref. ^2^. The sample size was calculated using power and sample size calculations program (Sealed Envelope Ltd. 2012). Twenty-six teeth were required to have a power of 80% and significance *α* = 5%. The sample size was increased to became 32 teeth with a dropout rate of 20%.

### Study setting

- The study was carried out in the Pediatric Dentistry and Dental Public Health Department, Faculty of Dentistry, Cairo University, Egypt.
- The procedures were carried out by a single postgraduate student having M.Sc. in Pediatric Dentistry, Faculty of Dentistry, Cairo University, Egypt.
- Dental unit: Knight by Midmark.
- X-ray machine: Minray, Soredex, Tuusula.

### Recruitment strategy

- A total of 32 vital first permanent molars with deep caries in 20 patients were selected randomly from the outpatient clinic of Pediatric Dentistry and Dental Public Health Department, Cairo University.
- Screening of patients continued until the target population was achieved.
- A written consent was obtained from all legal guardians.

### Inclusion criteria

- Patients free from any systemic diseases.
- Restorable permanent molars with deep carious lesions and at risk of pulp exposure if all caries is eliminated.
- The absence of swelling in periodontal tissues, fistula, and pathologic tooth mobility.
- The absence of clinical symptoms of irreversible pulpitis or sensitivity to pressure.
- The absence of any adverse radiographic findings as: radiolucencies at the interradicular or periapical regions or thickening of the periodontal spaces, absence of internal and external root resorption, absence of calcification in pulp tissue.
- Compliant patient/parent.

### Exclusion criteria

- Teeth previously restored.

### Randomization and allocation concealment

Patients were allocated randomly with 1:1 allocation ratio into two groups (*n* = 16) based on the capping material used. Group 1: PAD, Group 2: calcium hydroxide.

#### Sequence generation

Sequence generation was done for the patient number (1–32) using computer sequence generation (www.random.org). The sequence generator icon was selected from the home page, the sample size was stated, and two columns format was ordered. The result was then copied to group 1 and group 2 with randomized patients’ numbers (16 numbers in each group).

#### Allocation concealment

Each participant’s guardian withdrew a closed white envelope containing the sequence generation number before application of the corresponding material. Those closed white envelops included paper charts, which were folded eight times not to show its contents to assure allocation concealment.

#### Blinding

The child participants and legal guardian of each participating child, the radiographic outcome assessor, and the statistician were blind to whether it is an experimental or a control group.

### Intervention

#### Diagnostic procedure

The patients personal, medical, dental histories were taken from all patients participating in this study and recorded in a diagnostic chart. Clinical examination was then done by mirror and probe under good lighting condition, then a preoperative photograph and conventional periapical radiograph were taken. Consistent comparisons of the radiographs were possible using individual acrylic extension cone paralleling (XCP) index, which was prepared for each patient by registering the bite and placed around the XCP plastic tip.

### Clinical steps

(a) *Clinical steps for both groups*:

1. Administration of local anesthesia (mepivacaine hydrochloride 3% (Septodent®, Maurdes-Fosses, France).
2. Rubber dam isolation (Roeko rubber dam, a non-latex flexi dam (Coltène/Whaledent, Germany).
3. Opening of the cavity and the removal of undermined enamel using high speed handpiece with copious air/water spray and round burs (Komet, Germany and Dia-Burs, Mani, INC., Japan).
4. Complete removal of caries at the lateral walls of the cavity and at the dentino–enamel junction was done with excavators or low speed round burs.
5. Partial removal of carious dentin (only soft disorganized dentin) was removed on the pulpal wall.
6. Washing the cavity with distilled water and dryness with triple airway syringe and sterile cotton.
7. Closed white envelope was withdrawn by the child’s guardian to allocate the group.

(b) *Clinical steps for experimental group (photo activated oral disinfection)*:

1. Application of Aseptim solution (solution of dilute pharmaceutical grade tolonium chloride, water and sodium phosphate buffer supplied in the form of a syringe) using disposable tip and covering the whole cavity with agitation of the solution for 60 s using a brush.
2. Placement of the light disposable tip in the center of lesion and holding it just above the surface.
3. Activation of the red light for 60 s and holding the tip centered on the lesion.
4. After disinfection, the cavity was sealed immediately with glass ionomer (GC Fuji IX GP fast: (GC Co., Japan)) and then filled with composite resin (Voco x-tra fill a packable bulk fill posterior composite, Voco universal bond agent (VOCO, America) as final restoration.
5. Immediate postoperative radiograph was taken with size 2 Digora imaging plate and direct digital imaging was performed using the Digora system (Digora Optime, Soredex, Tuusula, Finland).

(c) *Clinical steps for comparative group (calcium hydroxide group)*:

1. Calcium hydroxide (Dycal® DENTSPLY Caulk a calcium hydroxide paste) was applied using calcium hydroxide applicator followed by glass ionomer and then composite resin as a final restoration as in experimental group.
2. Immediate postoperative radiographic was taken. with size 2 Digora imaging plate and direct digital imaging was performed using the Digora system (Digora Optime, Soredex, Tuusula, Finland).

### Outcomes

Evaluation was carried on 2, 6, 9, and 12 months for the following clinical and radiographic outcomes:

#### Primary outcome

- *Postoperative pain*: using Verbal rating scale.

#### Secondary outcomes

**Fig. 1:**
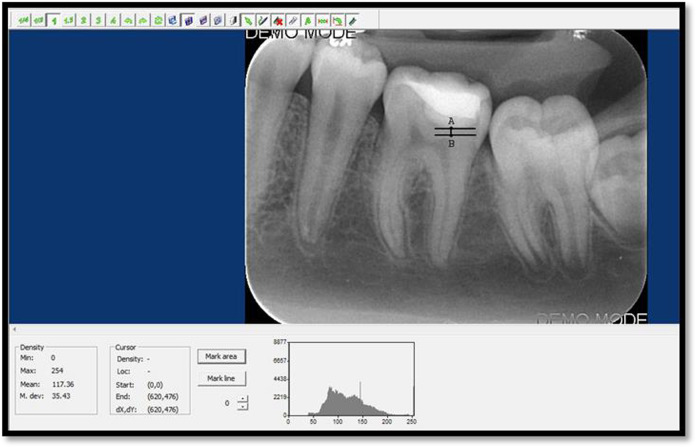
Photographs showing the measurement of the remaining dentin thickness on the digora software.

**Fig. 2:**
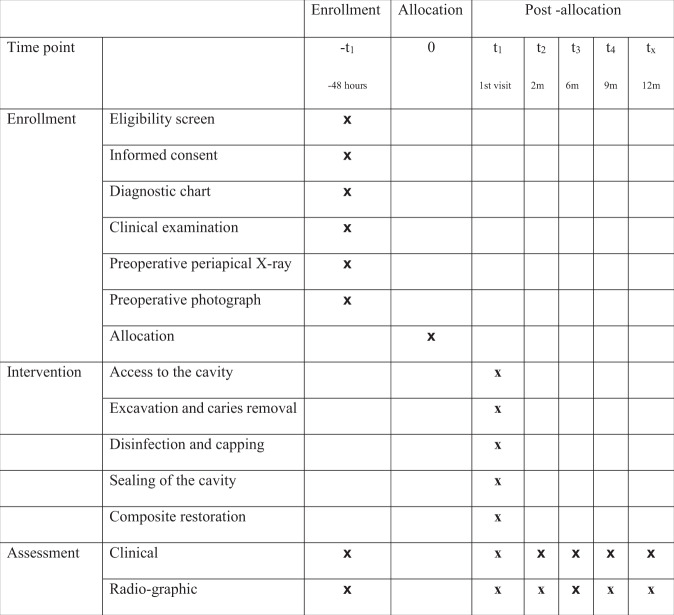
Photographs showing participant timeline.

- *Pain on percussion*: by tapping the tooth with the back of the mirror.
- *Swelling, Sinus or fistula*: by visual examination by the examiner.
- *Adverse radiographic findings*: Presence or absence of adverse radiographic findings was detected by two different radiographic examiners and inter-examiner reliability was calculated.
- *Thickness of newly formed dentin*: Newly formed dentin thickness was calculated after measuring the remaining dentin thickness using the Digora software (Digora for windows 2.5 software). For proper measurement, two defined tangential lines were drawn passing through two defined repeatable points (A and B) where A represents the deepest point of the restoration and B represents the deepest corresponding point in the dentin. A third line was drawn joining the two points together and measured in mm. This line represents the remaining dentin thickness and was measured in the immediate posttreatment and subsequent follow-up radiographs (2, 6, 9, and 12 months) to determine the newly formed dentin thickness by subtraction of the measurements of the immediate remaining dentin thickness from all follow-up periods according to Sharma et al.^9^ (Fig. 1). Participant timeline is shown in Fig. 2.

### Statistical analysis

Data was collected, checked, revised, tabulated, and entered into the computer. Quantitative variables from normal distribution were expressed as mean and standard deviation (SD) values. To test the significant differences between two groups at different follow-up periods, student *t* test was used. Significant level was set at *P* ≤ 0.05. Statistical analysis was done using the statistical package for social science (IBM®, SPSS® statistics for windows computer software version 20). The inter-examiner reliability was determined through calculation of Fleiss’ Kappa and percent of agreement using MedCalc® (statistical software version 16.8.4 for windows).

## Results

The present study included 32 vital permanent molars with deep caries in 20 healthy cooperative children that were selected randomly from the outpatient clinic of Pediatric Dentistry and Dental Public Health Department, Cairo University.

Children were divided into two groups: Group 1 was treated using PAD with a mean age 9.94 years and Group 2 was treated using calcium hydroxide with a mean age 9.75 years. There was an equal distribution of male and female in both groups with nine females and seven males per group (Table 1).

**Table 1:** Shows the distribution of the age and gender of the participants in each group.

	Age (years)				Gender		
	Mean	SD	Min.	Max	Female *N* (%)	Male *N* (%)	Total *N* (%)
Group I	9.94	1.731	7	12	9 (56.3)	7 (43.7)	16 (100)
Group II	9.75	1.653	7	12	9 (56.3)	7 (43.7)	16 (100)

**Fig. 3:**
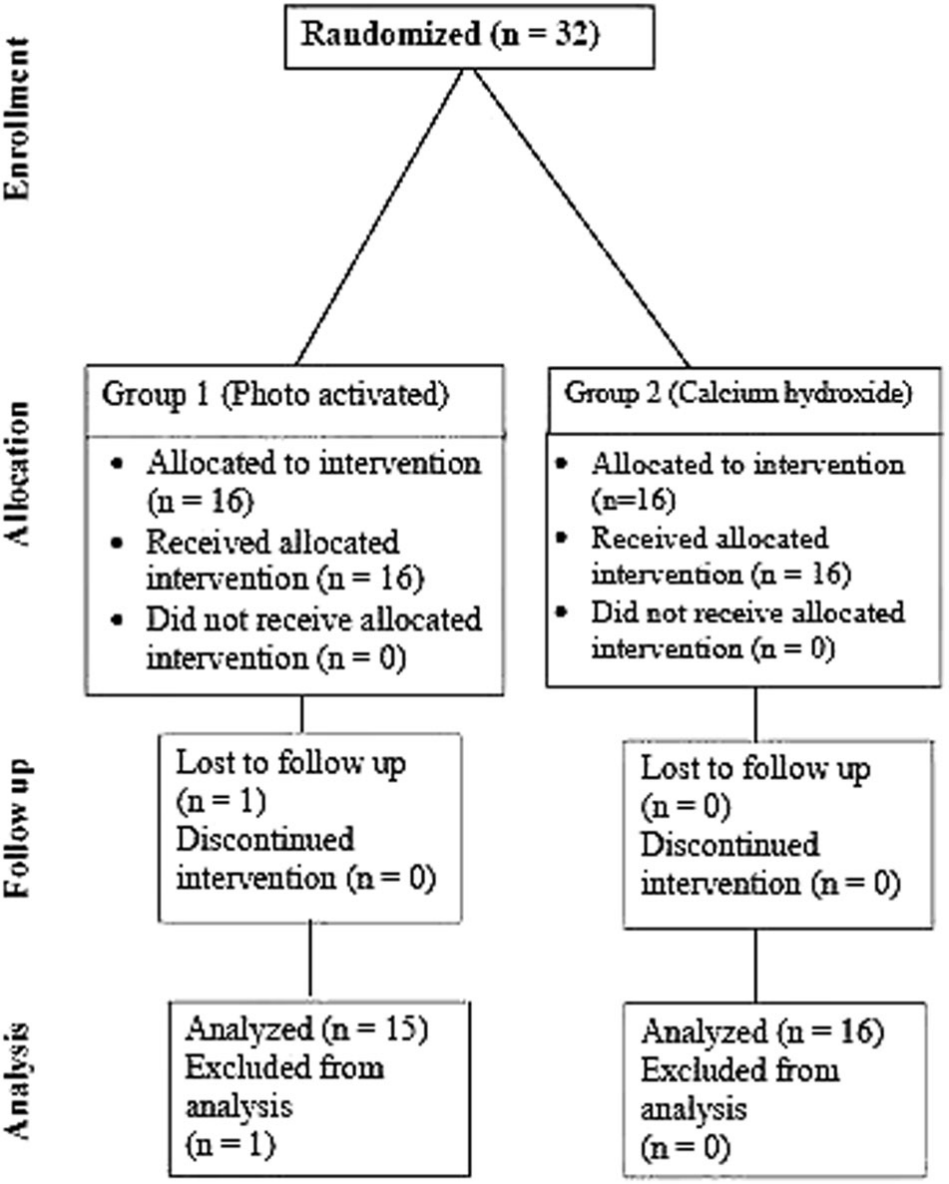
Flow chart showing the number of cases involved in the two groups at different study periods.

Cases were evaluated according to the clinical and radiographic criteria at 2, 6, 9, and 12 months. Figure 3 shows the number of cases involved in the two groups at different study periods.

Clinical success for both groups was 100% as there was absence of postoperative pain, swelling, pain on percussion, sinus or fistula for all follow-up periods (Table 2).

**Table 2:** Showing the clinical outcomes in both groups.

Clinical parameter	Results
Postoperative pain	Using the verbal rating scale, there was no postoperative pain in the two groups for all follow-up periods
Pain on percussion	There was no postoperative pain on percussion in the two groups for all follow-up periods
Swelling, sinus or fistula	There was no postoperative swelling sinus or fistula in the two groups for all follow-up periods

Radiographic success for both groups was also 100% as there was absence of adverse radiographic findings in both groups for all follow-up periods. Radiographic success was evaluated through the absence of adverse radiographic findings at entire follow-up periods. All radiographs were assessed blindly by the aid of two different evaluators at different times. The inter-examiner reliability was calculated using Fleiss’ Kappa with the percent of agreement. The inter-examiner reliability was found to be Kappa = 1 with 100% of agreement and 95% confidence interval (1.000 to 1.000).

Concerning the thickness of newly-formed dentin: The mean initial remaining dentin thickness for each group was 0.89 (±0.1) mm and 0.81 (±0.1) mm immediately after indirect pulp treatment for group 1 and group 2, respectively. There was no statistical significant difference between them with a *p* value = 0.087. Regarding the mean thickness of newly formed dentin for both groups at different follow-up periods, there was no statistical significant difference between both groups at 2, 6, 9, and 12 months, with *P* values = 0.825, 0.146, 0.280, and 0.400, respectively (Table 3).

**Table 3:** Showing the increase in the thickness of newly formed dentin in mm in each group at different follow-up periods.

	2 Months	6 Months	9 Months	12 Months
Group I	0.22 (±0.1)	0.43 (±0.16)	0.62 (±0.22)	0.70 (±0.30)
Group II	0.23 (±0.12)	0.35 (±0.16)	0.53 (±0.24)	0.62 (±0.33)

**Fig. 4:**
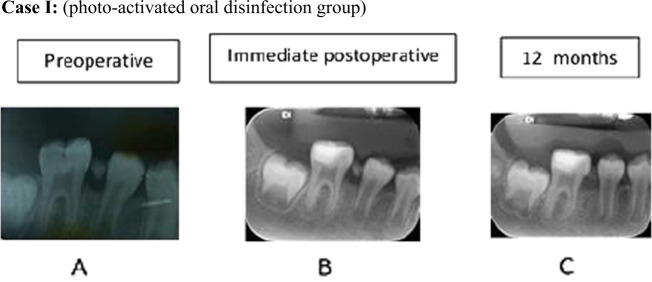
Photograph showing radiographic photos for a case from the photo-activated group at different stages. Photograph **a** showing preoperative radiographic photo **b** immediate postoperative radiographic photo **c** radiographic evaluation after 12 months.

**Fig. 5:**
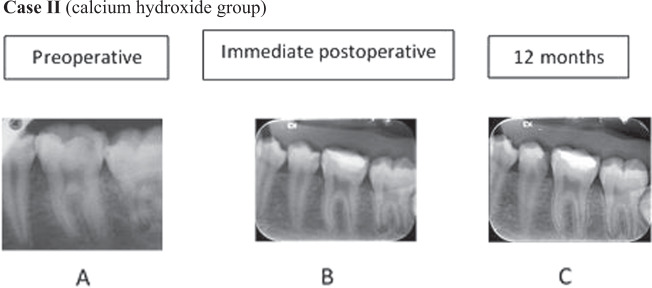
Photograph showing radiographic photos for a case from the calcium hydroxide group at different stages. Photograph **a** showing preoperative radiographic photo **b** immediate postoperative radiographic photo **c** radiographic evaluation after 12 months.

Cases presentation for Group 1 are shown in Fig. 4 and in Fig. 5 for Group 2.

## Discussion

There are many modes to preserve pulpal vitality in teeth with deep caries. Indirect pulp treatment is one such therapeutic modality that attempt to maintain pulp vitality and avoid more extensive treatments.^9^ The systematic review of Bergenholtz et al.^10^ showed that there are significant gaps in our knowledge concerning the treatment of the vital pulp with deep carious lesions and this study was carried out to compare two different methods for indirect pulp treatment.

In this study, 32 vital lower permanent first molars with deep caries in 20 children were involved and splitted up into two groups. Group 1 using PAD and group 2 using calcium hydroxide. All children were selected randomly from the outpatient clinic of Pediatric Dentistry and Dental Public Health Department, to unify the socioeconomic and educational levels. There was an equal distribution of the baseline characteristics as the two groups did not vary in socioeconomic and educational levels or demographic parameters and thus any systematic selection bias for the results of the study can be excluded. The high recall rate in the last follow-up period reduced the detection and reporting bias in this study.

Age selection in this study ranged from 6 to 12 years as in young patients, the excellent vascular supply, the presence of odontoblasts and undifferentiated mesenchymal cells, which are responsible of dentinogenetic function, favors tertiary dentin formation in deep carious lesions.^11^

Since there is no definite evidence that it is essential to reenter the tooth to remove the residual caries, single visit IPT was done for the two groups involved according to several studies using single visit IPT.^2,9,12,13^

Following pulp capping procedure, the cavity was sealed with glass ionomer and then filled with composite resin as final restoration. Sealing the demineralized dentin with a restoration that offers good peripheral seal and deprives the microorganisms from the oral cavity substrate. This reduces the bacterial count, diversity and arrests the caries process.^4^ Clinical and radiographic success for both groups were evaluated till one year according to comparable studies.^12–14^

Evaluation was carried on 2, 6, 9, and 12 months for the clinical and radiographic outcomes. Digital radiography was used with a very low dose and exposure time which is much lower than the corresponding regular radiography (nondigital periapical radiographs). Therefore, the digital radiographs taken were approximately equivalent to two regular periapical radiographs of D speed according to Carestream exposure timing reference manual.^12^ This was accepted by the research ethics committee of the Faculty which coincide with the country’s regulations. In addition, the children were protected with lead apron and paralleling technique with XCP index was used which ensured that the film is placed in proper position. Thus, no retakes were done.

Regarding the results of this study, the clinical and radiographic success for both groups was 100% at all follow-up periods. The good clinical and radiographic success reported here could be attributed to the correct diagnosis, complete removal of soft dentin and the use of a well-sealing coronal restoration that could prevent microleakage. These results were comparable to Gruythuysen et al.^13^ who reported a high survival rate of IPT done in primary and permanent teeth of young patients. Survival was defined as teeth without clinical or radiologic signs or symptoms. The study findings were also in agreement to Sharma et al.^14^ who compared the effectiveness of disinfection of remaining carious dentin in deep cavities with PAD against calcium hydroxide and found successful outcome for the treated teeth at 45 days, 6 months, and 12 months with only one tooth evidence of apical periodontitis, which was considered failure.

Concerning the mean thickness of newly-formed dentin for both groups at different follow-up periods. There was no statistically significant difference between both groups at 2, 6, 9, and 12 months, with *P* values = 0.825, 0.146, 0.280, and 0.400, respectively. This might be due the absence of differences between both groups starting from baseline characteristics, case selection to the technique performed. Also, the sample used in this study might be considered as a factor for this finding.

The results were in accordance to Leye Benoist et al.^2^ which assessed the effectiveness of mineral trioxide aggregate compared to calcium hydroxide as an indirect pulp-capping material in molar and premolar teeth. The mean initial residual dentin thickness increased at 3 and 6 months in calcium hydroxide group. Also. Sultana et al.^15^ compared mineral trioxide aggregate and calcium hydroxide as capping materials. They showed that at 3 months, reparative dentin was formed in 17 teeth (of 25 teeth) in the calcium hydroxide group and at 6 and 12 months, 19 treated teeth showed evident of reparative dentin formation.

These results were opposite to de Pinheiro et al.^16^ who reported no increase or decrease in remaining dentin thickness after 12 months when compared with the baseline. This difference in findings could be justified by the presence of some difficulties such as precise measurement of lesion depth. The clinical carious lesion might be deeper than the assumed lesion’s radiographic appearance which makes the determination of the remaining dentin thickness clinically and radiographically difficult.^17^ The possible limitations of this study include the small sample size and the deficiency of clinical studies on the use of PAD.

## Conclusions

The clinical and radiographic success for indirect pulp treatment of young permanent molars with both PAD and calcium hydroxide were comparable. Further studies with larger sample sizes and different materials and techniques for IPT are recommended.

### Why this paper is important to pediatric dentistry?

Based on the results of this study,

- Indirect pulp capping can be used in the treatment of deep carious lesions with a good success rate.
- Proper diagnosis of the pulpal conditions and a well-sealing restoration aided in the success of IPT.
- PAD and calcium hydroxide reported equal effect in the treatment of deep carious dentin.

## References

[1] American Academy of Pediatric Dentistry. (2015). Guideline on pulp therapy for primary and immature permanent teeth. Ref. Man. Pediatr. Dent..

[2] Leye Benoist F, Gaye Ndiaye F, Kane AW, Benoist HM, Farge P (2012). Evaluation of mineral trioxide aggregate (MTA) versus calcium hydroxide cement (Dycal(®)) in the formation of a dentine bridge: a randomised controlled trial. Int Dent. J..

[3] Jain, P. & Raj, J. D. (2015). Dentin substitutes: a review. Int. J. Pharma Bio Sci..

[4] Ricketts D, Lamont T, Innes N, Kidd E, Clarkson J (2013). Operative caries management in adults and children. Cochrane Database Syst. Rev..

[5] Hesse D (2014). Sealing versus partial caries removal in primary molars: a randomized clinical trial. BMC Oral. Health.

[6] Bonsor SJ, Pearson GJ (2006). Current clinical applications of photo-activated disinfection in restorative dentistry. Dent. Update.

[7] Patel Z. Photo activated disinfection in dentistry. *Eur. J. Pharm. Med. Res.* www.ejpmr.com.

[8] Nammour S (2010). Evaluation of dental pulp temperature rise during photo-activated decontamination (PAD) of caries: an in vitro study. Lasers Med. Sci..

[9] Hilton TJ (2009). Keys to clinical success with pulp capping: a review of the literature. Oper. Dent..

[10] Bergenholtz G (2013). Treatment of pulps in teeth affected by deep caries—a systematic review of the literature. Singap. Dent. J..

[11] Monea M, Stoica A, Monea A (2014). Clinical aspects: histological evaluation of indirect pulp capping procedures with calcium hydroxide and mineral trioxide aggregate. Acta Med. Transilv..

[12] Dental Carestream. Dental radiography series brochure.© Carestream Health. 2016:1–16.

[13] Gruythuysen RJM, Gruythuysen R, van Strijp AJP, van Strijp G, Wu M-K (2010). Long-term survival of indirect pulp treatment performed in primary and permanent teeth with clinically diagnosed deep carious lesions. J. Endod..

[14] Sharma S, Logani A, Shah N (2014). Comparative efficacy of photo-activated disinfection and calcium hydroxide for disinfection of remaining carious dentin in deep cavities: a clinical study. Restor. Dent. Endod..

[15] Sultana R, Hossain M, Alam S (2016). Evaluation of clinical and radiological outcomes of mineral trioxide aggregate and calcium hydroxide as indirect pulp capping agents in the treatment of deep carious lesion of permanent teeth. Bangabandhu Sheikh Muji Med. Univ. J..

[16] de Assunção Pinheiro IV, Borges BCD, de Lima KC (2012). In vivo assessment of secondary caries and dentin characteristics after traditional amalgam restorations. Eur. J. Dent..

[17] Orhan AI, Oz FT, Ozcelik B, Orhan K (2008). A clinical and microbiological comparative study of deep carious lesion treatment in deciduous and young permanent molars. Clin. Oral. Investig..

